# Spindle Epithelial Tumor With Thymus-Like Elements: A Case Report

**DOI:** 10.7759/cureus.79035

**Published:** 2025-02-15

**Authors:** Maheen Maruf, Sajid Mushtaq, Umer Sheikh

**Affiliations:** 1 Pathology, Shaukat Khanum Memorial Cancer Hospital and Research Centre, Lahore, PAK

**Keywords:** epithelial, rare, spindle epithelial tumor with thymus-like elements (settle), synovial sarcoma, thyroid

## Abstract

Spindle epithelial tumor with thymus-like elements (SETTLE) presents as a neck swelling in children and adolescents. It is a rare malignant neoplasm derived from ectopic thymus or branchial pouch derivatives. It has a propensity for late hematogenous metastasis. Morphologically, it is a biphasic tumor composed of fascicles of spindle cells and epithelial components in the tubulo-papillary pattern. Due to its rarity, pathologists often misdiagnose it. Synovial sarcoma is its main morphologic mimic, but SETTLE lacks t(X;18) translocation. Surgery is the mainstay of treatment. Radiotherapy can be offered for local recurrence and chemotherapy for distant metastases. We report a case of gradually increasing left thyroid swelling in a boy from Pakistan. His thyroid function tests were normal. Cytology showed a spindle cell lesion, Bethesda category 5. The patient underwent a total thyroidectomy. The left thyroid lobe showed a partially encapsulated, tan-white, firm, homogenous nodule. Histology showed an encapsulated, focally infiltrating tumor, composed of spindle and epithelial cells in lobules with mild atypia. Based on immunohistochemistry, medullary carcinoma and carcinoma with thymus-like elements (CASTLE) were ruled out. Molecular analysis for t(X;18) (p11.2; q11.2) ruled out synovial sarcoma. Therefore, the final diagnosis was SETTLE. The patient died at 42 months of follow-up due to local recurrence and lung metastases. SETTLE is a rare childhood thyroid neoplasm with late metastatic potential. Early diagnosis, surgical resection, and surveillance can ensure disease-free survival. Diagnosed cases should be reported to help formulate effective chemotherapy and radiotherapy regimens for the treatment of recurrence or metastasis.

## Introduction

Spindle epithelial tumor of thymus-like elements (SETTLE) is a rare thyroid neoplasm, accounting for a small fraction of pediatric thyroid malignancies with fewer than 100 reported cases in the literature [1]. SETTLE presents as a swelling in the neck in children. It was described by Chan and Rosai as a low-grade neoplasm derived from ectopic thymus or branchial pouch remnants [1,2]. Its main morphologic mimic is synovial sarcoma, but it lacks its t(X;18) translocation [3]. Histologically, it is a biphasic tumor composed of spindle cells in fascicles and epithelial cells in tubulo-papillary patterns. SETTLE exhibits a slow clinical course but has a significant risk of distant hematogenous metastasis, often occurring after several years of primary diagnosis with a median of 10 years [4,5]. It has good overall survival. Surgery is the mainstay of treatment. Approximately 50 cases have been reported in the literature [6]. However, cytologic features of SETTLE have rarely been described with histologic correlation [2,7]. Only one study has been reported previously from Pakistan on the basis of histology [8].

This report presents a comprehensive evaluation of SETTLE in a pediatric patient from Pakistan, detailing cytologic, histologic, immunohistochemical, and molecular findings along with treatment outcomes. In addition, we review existing literature to highlight diagnostic challenges and therapeutic considerations.

## Case presentation

**Figure 1:**
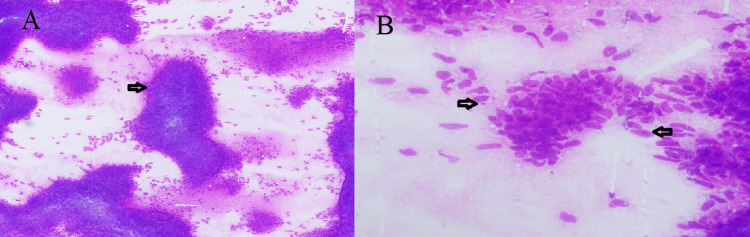
Cytology smears A. Moderately cellular smears with cohesive clusters (arrow H&E10X). B. Ovoid to spindled cells with a high nuclear-cytoplasmic ratio (arrows H&E 40X).

A nine-year-old boy from Pakistan presented with left thyroid swelling for three years, gradually increasing in size and painful during coughing. He appeared well and his thyroid function tests were normal. Fine-needle aspiration cytology (FNAC) showed moderate cellular smears with cohesive ovoid to spindled cells with a high nuclear-cytoplasmic ratio and bland hyperchromatic nuclei with powdered chromatin. The cytology findings suggested a spindle cell neoplasm, Bethesda category V, raising suspicion of medullary carcinoma of the thyroid. It was ruled out due to negative chromogranin and synaptophysin performed on the cell block and normal serum calcitonin levels (Figure 1).

The patient underwent total thyroidectomy with selective neck dissection at a local hospital. No intraoperative frozen section was performed. The specimen was received at our hospital laboratory in 10% buffered formalin, paraffin-embedded, and routinely sectioned for histological evaluation. The right thyroid lobe measured 4.8 x 1.8 x 1.7cm, weighing 6 grams, and was grossly unremarkable. The left thyroid lobe measured 9.6 x 7 x 4.5 cm, weighing 110 grams. Gross examination revealed a well-demarcated, partially encapsulated tumor measuring 9.5 x 6.5 x 3 cm with focal capsular penetration but no definitive extrathyroidal extension or necrosis.

**Figure 2:**
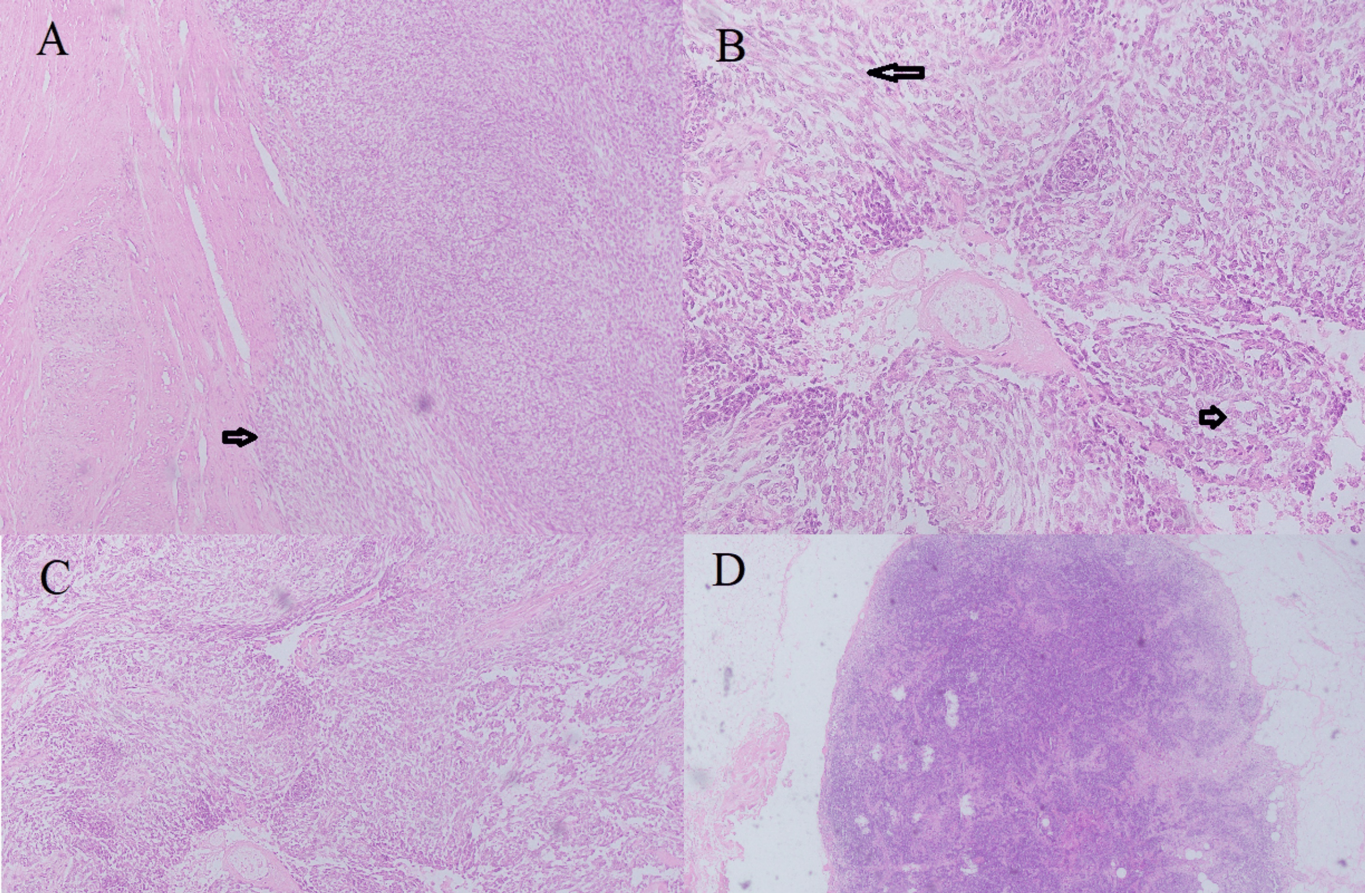
Histology of thyroid tumor A. Encapsulated, focally infiltrating tumor with lobular architecture (arrow H&E 10X). B. Spindle cells with scant cytoplasm, elongated nuclei, and fine chromatin in fascicles (longer arrow) and epithelial cells are cuboidal forming tubules (smaller arrow) with mild to moderate atypia (H&E 20X). C. No necrosis seen (H&E 10X). D. Lymph node negative for metastasis (H&E 10X).

Histology revealed an encapsulated, focally infiltrating tumor with lobular architecture. The lobules were separated by sclerotic stroma, composed of spindle cells in fascicles and epithelial cells forming tubules with mild to moderate atypia, 3-4 mitosis/10 HPF, and absent necrosis. The lymph nodes were negative for metastasis (Figure 2).

**Figure 3:**
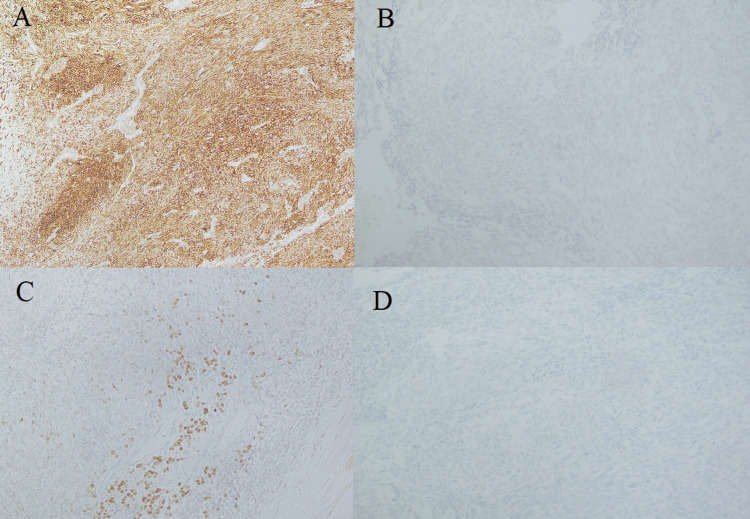
Immunohistochemical panel A. CK is positive (cytoplasmic staining) in tumor cells. B. Synaptophysin is negative in tumor cells. C. TTF1 is negative in tumor cells , nuclear staining in few residual follicles. D. Desmin is negative in tumor cells.

The morphological differential diagnosis was medullary carcinoma, carcinoma with thymus-like elements (CASTLE), spindle epithelial tumor with thymus-like elements (SETTLE), and synovial sarcoma. The tumor exhibited epithelial differentiation, evidenced by CK and CAM 5.2 positivity. Focal EMA positivity, although unusual, has been reported in SETTLE. Importantly, negative staining for thyroid transcription factor (TTF1), calcitonin, and synaptophysin helped exclude medullary carcinoma and CASTLE. The absence of S-100 ruled out ectopic hamartomatous thymoma, anaplastic carcinoma is rare in children, and the lack of atypia, mitoses, and necrosis excluded it. The lack of t(X;18) translocation on fluorescence in-situ hybridization (FISH) confirmed the diagnosis of SETTLE over synovial sarcoma. The patient developed lung nodules at four months follow-up; however, he did not pursue further treatment. He died at 42 months of follow-up due to local recurrence and metastases (Figure 3).

## Discussion

SETTLE is a rare thyroid neoplasm. It usually presents as a growing thyroid nodule in children and young adults for a variable period [1,9]. Their mean age was 19 years with a range of two to 59 years [2]. The tumor has slight male predilection with the male-to-female ratio being 1.5:1 [9,10] as seen in our case as well. Usually, the right lobe of the thyroid is involved [10], and rarely, the entire gland is rarely replaced by a hard tumor, simulating thyroiditis [1]. However, our case presented with unilateral left-sided, firm swelling. This raises the differential diagnosis of goiter, differentiated epithelial thyroid tumors, medullary carcinoma, and spindle cell tumors. No predisposing factors of iodine deficiency or exposure to radiation have been reported [9].

Although SETTLE is a well-documented entity histologically, its cytologic features remain underreported, leading to frequent misdiagnosis [9]. It should be included in differential diagnosis along with medullary carcinoma and synovial sarcoma if spindle cell lesions are encountered in this age group on cytology. The smears of medullary carcinoma reveal a mixture of spindle and plasmacytoid epithelial cells with amyloid in the background. While synovial sarcoma reveals severe cytologic atypia, numerous mitoses, and necrosis on cytology [2]. It can be misdiagnosed as papillary carcinoma of the thyroid (PTC) as well, but SETTLE lacks PTC nuclear features and TTF1 positivity [11]. Low familiarity with this entity makes its diagnosis challenging.

SETTLE does not have any unique clinical features, imaging findings, or tumor markers; therefore, histopathologic evaluation is the gold standard for diagnosis [1]. Histologically incomplete lobulations, biphasic epithelial and spindle cell populations, and cleft-like spaces can be seen [2]. The light microscopic differential diagnosis of SETTLE includes many spindle and epithelial tumors. The ectopic hamartomatous thymoma can be distinguished by age, site, and intermixed adipocytes. The ectopic cervical thymus has scattered lymphocytes and usually occurs in older age groups. Carcinomatous and immature mesenchymal elements are differentiating features from the teratoma of the thyroid gland. The spindle cell variant of medullary carcinoma differs by amyloid deposits and calcitonin staining [8].

Immunohistochemistry aids in excluding other diagnoses. The literature from India, South Korea, and Brazil shows that SETTLE usually shows expressions for CK, S-100, SMA, BCL2, and CD99 and negative expression for EMA, CEA, CK19, calcitonin, thyroglobulin, and chromogranin immunohistochemical stains [1,2,12]. Another case report from Africa showed positivity for CK, galectin-3, and HBME but was negative for CEA, S-100, CD 31, CD 34, chromogranin, calcitonin, p53, and CD117 [10]. Our case also showed positive expression for CK, CAM 5.2, and CKHMW and negativity for synaptophysin, thyroglobulin, and calcitonin, as shown in Figure 3. There was unusual positivity for EMA and negativity for S-100 in our case. A judicious panel of immunohistochemical stains, clinical correlation, and a high index of suspicion can help distinguish SETTLE from its mimics.

There are two mimickers of SETTLE after immunohistochemistry. The ectopic cervical thymoma may involve the whole gland. Synovial sarcoma of the head and neck is composed of monomorphic spindle cells, hyperchromatic nuclei, mild pleomorphism, and mitoses [8]. Cytokeratin is patchy positive in synovial sarcoma and diffuse positive in SETTLE. EMA is positive in synovial sarcoma and negative in SETTLE, as seen in our case as well. Synovial sarcoma shows t(X;18) (p11.2; q11): SYT-SSX1 fusion, which is absent in SETTLE [3,8] like in our case. This confirmed the diagnosis of SETTLE. No hallmark genetic mutation has been described for SETTLE so far [6].

Surgery is the main mode of treatment, and close follow-up should be done [11,13]. Only a few cases undergo neck dissection as well [9]. SETTLE has an overall survival of 86% (median follow-up of six years)[5]. Its metastatic rate is 71% in patients with more than five years of follow-up, despite its indolent course [4]. It usually metastasizes to the lungs, cervical lymph nodes, kidneys, and delayed hematogenous metastasis [8]. Renal metastases have poor outcomes [14]. The tumor retains its original morphology in metastases [12]. In our case, the child developed lung nodules and local recurrence and died at 42 months after surgery. There is one other case reported from Pakistan in which a child remained disease-free after 64 months of follow-up unlike our study [8]. In many other cases, the patient was alive and free of recurrence on 30, 41, 64, and 72 months after surgery [8,10,13]. These contrary results in our case might be due to low socioeconomic status, difficulty seeking appropriate timely medical help, and appropriate knowledge of this entity.

There is no definitive treatment protocol for metastatic SETTLE due to a limited number of patients. However, the literature shows that a combination of different lines of chemotherapy, anti-EGFR antibodies, and radiotherapy to localized sites can prolong disease control [15]. Despite metastasis, it shows good overall survival with treatment [7]. Single studies show KRAS and KMT2D gene mutations in SETTLE. It lacks t(X;18) and RET mutations, differentiating it from synovial sarcoma and spindle cell variants of medullary thyroid carcinoma, respectively [3]. However, additional molecular studies are required to discover the underlying genetic mechanisms of this rare entity.

## Conclusions

SETTLE is an uncommon childhood thyroid neoplasm with metastatic potential. Cytology alone can misdiagnose it. Histology is the gold standard for diagnosis. Surgery is the mainstay of treatment. Radiotherapy can be given for local recurrence and chemotherapy for distant metastases. Long-term follow-up is essential to detect late metastases.

This is the first comprehensive study of SETTLE from Pakistan with cytological, molecular, histological, and prognostic correlations. Diagnosed cases should be reported to help formulate effective chemotherapy and radiotherapy regimens for the treatment of recurrence or metastasis. Knowledge of this entity, early diagnosis, complete surgical resection, and surveillance can ensure disease-free survival. Additional molecular studies are warranted to unravel underlying genetic mechanisms for potential targets for immune therapy.
